# Detection and Genetic Characterization of Deltacoronavirus in Pigs, Ohio, USA, 2014

**DOI:** 10.3201/eid2007.140296

**Published:** 2014-07

**Authors:** Leyi Wang, Beverly Byrum, Yan Zhang

**Affiliations:** Ohio Department of Agriculture, Reynoldsburg, Ohio, USA

**Keywords:** genetic characterization, deltacoronavirus, Deltacoronavirus genus, Coronaviridae, pigs, sows, piglets, diarrheal disease, pig farms, Ohio, United States, viruses

## Abstract

In Ohio, United States, in early 2014, a deltacoronavirus was detected in feces and intestine samples from pigs with diarrheal disease. The complete genome sequence and phylogenetic analysis of the virus confirmed that the virus is closely related to a porcine deltacoronavirus (porcine coronavirus HKU15) reported in Hong Kong in 2012.

Coronaviruses (order *Nidovirales*, family *Coronaviridae*, subfamily *Coronavirinae*) are single-stranded, positive-sense, enveloped RNA viruses with a genome size ranging from 25.4 to 31.7 kb (*1*). Coronaviruses traditionally were classified into groups 1, 2, and 3 on the basis of their antigenic relationships (*2*). The traditional classification recently was replaced by 4 genera (*Alphacoronavirus*, *Betacoronavirus*, *Gammacoronavirus*, and *Deltacoronavirus*), as described by the International Committee for Taxonomy of Viruses (http://www.ictvonline.org/virusTaxonomy.asp?msl_id = 27). Virus from each coronavirus genus has been found in diverse host species, including mammals and birds (*3*). Viruses of the *Alphacoronavirus*, *Betacoronavirus*, and *Deltacoronavirus* genera have been detected in swine (*3*). Transmissible gastroenteritis virus and porcine epidemic diarrhea virus (PEDV) are members of the genus *Alphacoronavirus*; these viruses cause severe diarrhea in swine herds, leading to significant economic loss in many countries, including the United State (*4*–*6*). There is little information about deltacoronavirus infections in pigs, and only 1 surveillance study from Hong Kong reported its detection in pigs (*1*). The virus has not been reported to be associated with clinical disease in these animals. We report the detection and genetic characterization of a deltacoronavirus in pigs from farms in Ohio, United States; the pigs all had clinical diarrheal disease.

## The Study

During the end of January and the beginning of February in 2014, feces and intestine samples from pigs on 5 Ohio farms were submitted to the Animal Disease Diagnostic Laboratory at the Ohio Department of Agriculture. The farm managers reported outbreaks of diarrheal disease in sows and piglets. The clinical signs were similar to those associated with PEDV infection, including watery diarrhea in sows and death in piglets. However, the death rate in piglets (30%–40%) was lower than that typically observed with PEDV infection. Test results for samples from Farm 1 were negative for PEDV, transmissible gastroenteritis virus, rotavirus, and *Salmonella* spp. Examination of the samples by electron microscopy showed that most contained coronavirus-like virus particles. This finding prompted the laboratory to look further for the presence of viruses other than alphacoronaviruses.

Because deltacoronavirus has been reported in pigs (*1*), we designed a deltacoronavirus-specific reverse transcription PCR (RT-PCR). RNA was extracted from feces and intestine samples by using the TRIzol (Invitrogen, Carlsbad, CA, USA) method, and RT-PCR was performed by using the QIAGEN OneStep RT-PCR kit (QIAGEN, Hilden, Germany) with porcine deltacoronavirus–specific primers for membrane (M) gene 67F(5′-ATCCTCCAAGGAGGCTATGC-3′), 560R(5′-GCGAATTCTGGATCGTTGTT-3′), and nucleocapsid (N) gene 41F(5′-TTTCAGGTGCTCAAAGCTCA-3′), 735R(5′-GCGAAAAGCATTTCCTGAAC-3′). These primers were designed by using the conserved regions of 2 available porcine deltacoronavirus sequences (*1*). RT-PCR was performed under the following cycling conditions: 50°C for 30 min and 95°C for 15 min for the RT reaction, followed by 40 cycles of amplification at 95°C for 15 s, 55°C for 45 s, and 72°C for 1 min, with a final extension at 72°C for 7 min. All samples (3 piglet small intestines and 9 feces samples) from Farm 1 were positive for deltacoronavirus by the 2 RT-PCR assays for M and N genes (Table 1). Nucleotide sequences were determined for both amplified M and N fragments. A BLAST (http://blast.ncbi.nlm.nih.gov/Blast.cgi) search of the sequences of both M and N fragments showed 99% nt identity with the porcine coronavirus HKU15 (PorCoV HKU15)-155. Therefore, the virus detected in this study was PorCoV HKU15, which belongs to the *Deltacoronavirus* genus.

**Table1:** Detection of porcine coronavirus HKU15 and porcine epidemic diarrhea virus in samples from pigs on 5 farms in Ohio, USA, 2014*

Farm no	No. samples positive/total samples tested for		
	Porcine coronavirus HKU15		Porcine epidemic diarrhea virus
1	12/12†		0/12
2	8/11‡		2/11
3	8/8		1/8
4	4/4		1/4
5	7/7		1/7

*Porcine coronavirus HKU15 was detected by reverse transcription PCR. Porcine epidemic diarrhea virus was detected by real-time reverse transcription PCR. The samples consisted of 42 feces and intestine samples from pigs with diarrheal disease.  †Of the positive samples, 3 were from piglets. ‡Of the positive samples, 2 were from piglets.

RT-PCR was then run on samples from Farms 2–5. The results from the 5 farms are summarized in Table 1. Of the total 42 samples from the 5 farms, 39 (92.9%) were positive for PorCoV HKU15 by RT-PCR (Table 1). In addition, 5 (11.9%) of the 42 samples were positive for classical US PEDV instead of a recently reported variant PEDV (*7*) (Table 1). None of the samples tested were positive for transmissible gastroenteritis virus. Four (9.5%) of the 42 samples were positive for PorCoV HKU15 and PEDV, indicating that mixed infection with PorCoV HKU15 and PEDV occurred in some pigs.

On the basis of 2 complete genome sequences from GenBank, of PorCoV HKU15-44 and HKU15-155, we designed 16 pairs of primers to determine the whole genome of extracted RNA samples (OH1987) from Farm 1 (Table 2). The genome of PorCoV HKU15 OH1987 comprised 25,422 nt (GenBank accession no. KJ462462). The genome organization and the transcription regulatory sequence motif 5′-ACACCA-3′ of PorCoV HKU15 OH1987 are the same as those reported for PorCoV HKU15-155 (*1*). Similar to the BLAST search results of partial N and M fragments, the BLAST search of the whole genome of the PorCoV HKU15 OH1987 showed 99% nt identity to PorCoV HKU15-155. BLAST search of the spike gene of PorCoV HKU15 OH1987 showed 99% nt identity to PorCoV HKU15-44. These results confirmed that the coronavirus detected was a deltacoronavirus.

**Table 2:** Oligonucleotide primers used for amplification of the porcine coronavirus HKU15 genomic fragments by reverse transcription PCR, Ohio, USA, 2014

Primer identification	Sequence (5′→3′)	Nucleotide position*	Fragment
Dcor-1-F	ACATGGGGACTAAAGATAAAAATTATAGC	1–29	1
DCor-1610-R	AGACGGGCCAATTTTGACCG	1591–1610	
DCor-1481-F	TGATGATGTT CTGCTAGCCT	1481–1500	2
DCor-3300-R	GCTCATCGCCTACATCAGTA	3281–3300	
DCor-3091-F	CGGATTTAAAACCACAGACT	3091–3110	3
Dcor-4860-R	ACGACTTTACGAGGATGAAT	4841–4860	
DCor-4741-F	CTCCTGTACAGGCCTTACAA	4741–4760	4
DCor-6420-R	TCACACGTATAGCCTGCTGA	6401–6420	
DCor-6291-F	CTCAATGCAGAAGACCAGTC	6291–6310	5
DCor-8041-R	CAGCTTGGTCTTAAGACTCT	8041–8060	
DCor-7920-F	GGTACTGCTTCTGATAAGGAT	7920–7940	6
DCor-9660-R	TAGGTACAGTTGTGAACCGA	9641–9660	
DCor-9541-F	CTCTGCCCATTATCATGCCT	9541–9560	7
DCor-11040-R	AAAGAGAGGCATTTTGCTGG	11021–11040	
DCor-10861-F	ACTTGGACCCTCCTATGCGC	10861–10880	8
DCor-12840-R	GGCTCAAGATACTTATCTGC	12821–12840	
DCor-12721-F	TATGCAGGATGGTGAAGCGG	12721–12740	9
DCor-14400-R	TCACAATAAATCGCAGTGCC	14381–14000	
DCor-14281-F	TGTTACGCAGACTACACATA	14281–14300	10
DCor-16020-R	TCATAGCCGCAGCGCTTAAA	16001–16020	
DCor-15901-F	TGTGGTGTTTAGGCAGGCAA	15901–15920	11
DCor-17760-R	GTGGCGGTTACGCCTAAACC	17741–17760	
DCor-17641-F	CAAACTCTTTGACAATCGCA	17641–17660	12
DCor-19200-R	GCTAAAGGAGAATAGGTTGGTG	19179–19200	
DCor-18981-F	CTGAACATTCATTCTCACCC	18981–19000	13
DCor-20910-R	GAAGGTGGTGGCATTTGTGG	20891–20910	
DCor-20761-F	GTCTTACCGTGTGAAACCCC	20761–20780	14
DCor-22440-R	AACATCCCACTGAGGAGGTG	22421–22440	
DCor-22321-F	TTTTATAACACCACCGCTGC	22321–22340	15
DCor-24004-R	GGCCATGATAGATTGGTGTC	23985–24004	
DCor-23881-F	ATGGTGAGCCTTTACTGCTT	23881–23900	16
DCor-25417-R	TGCTCCATCCCCCCTATAAG	25398–25417	

*Positions correspond to porcine coronavirus HKU15-155 strain (GenBank accession no. JQ065043).

**Figure 1:**
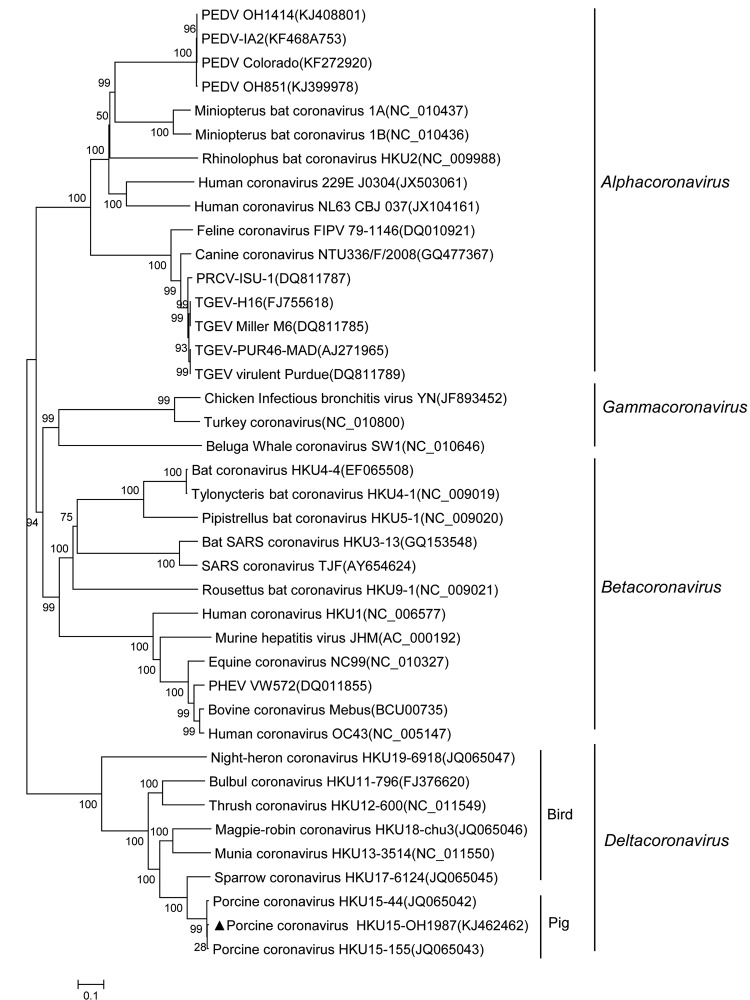
Phylogenetic tree constructed on the basis of the whole-genome sequences of virus strains from 4 coronavirus genera (*Alphacoronavirus*, *Betacoronavirus*, *Gammacoronavirus*, and *Deltacoronavirus*), including the porcine coronavirus HKU15 OH1987 strain (indicated with triangle). The dendrogram was constructed by using the neighbor-joining method in the MEGA software package, version 6.05 (http://www.megasoftware.net/). Bootstrap resampling (1,000 replications) was performed, and bootstrap values are indicated for each node. Reference sequences obtained from GenBank are indicated by strain name and accession number. Scale bar represents 0.1 nt substitutions per site. PEDV, virus and porcine epidemic diarrhea virus; PRCV, porcine respiratory coronavirus; TGEV, transmissible gastroenteritis virus; SARS, severe acute respiratory syndrome. PHEV, porcine hemagglutinating encephalomyelitis virus.

**Figure 2:**
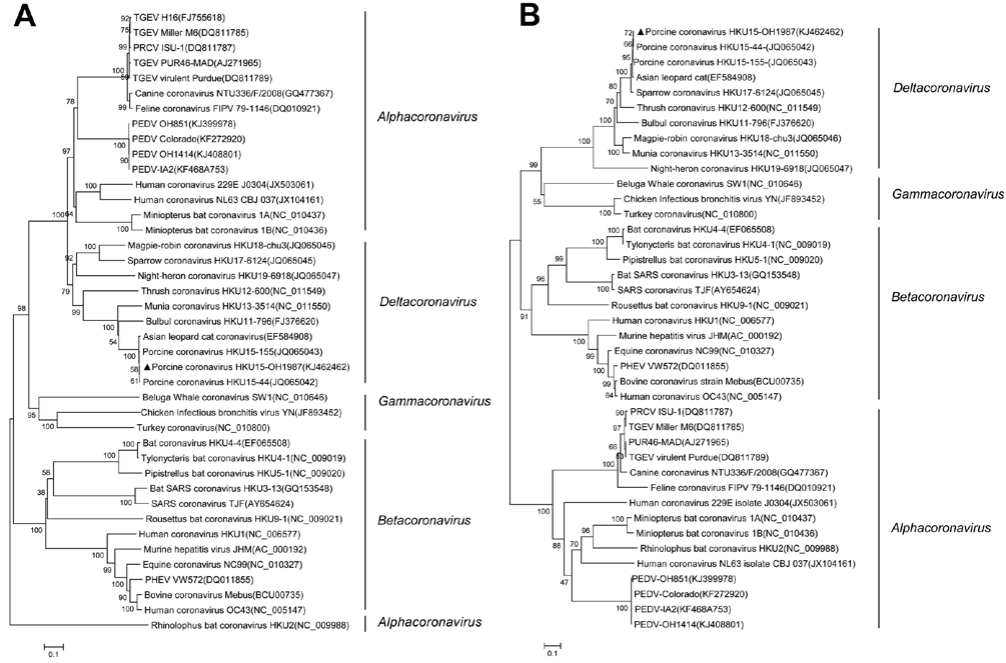
Phylogenetic analyses of spike protein (A) and nucleocapsid protein (B) of virus strains of 4 coronavirus genera (*Alphacoronavirus*, *Betacoronavirus*, *Gammacoronavirus*, and *Deltacoronavirus*), including the porcine coronavirus HKU15 OH1987 strain (indicated with triangle). The dendrogram was constructed by using the neighbor-joining method in the MEGA software package, version 6.05 (http://www.megasoftware.net/). Bootstrap resampling (1,000 replications) was performed, and bootstrap values are indicated for each node. Reference sequences obtained from GenBank are indicated by strain name and accession number. Scale bars represent 0.1 aa substitutions per site. PEDV, virus and porcine epidemic diarrhea virus; PRCV, porcine respiratory coronavirus; TGEV, transmissible gastroenteritis virus; PHEV, porcine hemagglutinating encephalomyelitis virus; SARS, severe acute respiratory syndrome.

Phylogenetic analysis of the complete genome of PorCoV HKU15 OH1987 showed that the OH1987 strain clustered with the other 2 PorCoVs, HKU15-155 and HKU15-44, and was distinct from the bird deltacoronaviruses (Figure 1). In addition, the phylogenetic trees constructed by using the amino acid sequences of the spike glycoprotein and nucleocapsid protein showed that the OH1987 virus clustered with HKU15-155 and HKU15-44 (Figure 2); this finding is in agreement with that in a previous study (*1*). The OH1987 virus differs from HKU15-155 in the spike gene at nt 19469 and in the noncoding region at nt 25044; a 3-nt insertion is present at each location, making the whole-genome sequence of the OH1987 virus 6 nt longer than that of HKU15-155. The 2 insertion sites of the virus are identical to those of the HKU15-44 strain of porcine deltacoronavirus. However, the genome of OH1987 virus is 1 nt longer than that of HKU15-44 because of the insertion site at nt 25263, located in 3′ untranslated region. Of interest, strain OH1987 was most closely related to HKU15-155 when whole-genome sequence was used for phylogenetic analysis (Figure 1), but strain OH1987 was more closely related to HKU15-44 when phylogenetic analysis of spike protein and nucleocapsid protein was performed (Figure 2). On basis of the partial genome sequence, strain OH1987 was also closely related to a deltacoronavirus found in the Asian leopard cat (the whole-genome sequence is not available in GenBank).

## Conclusions

We detected a porcine deltacoronavirus in pigs in the United States. Although the genetic and phylogenetic analyses showed that the newly emergent strain, PorCoV HKU15 OH1987, was closely related to 2 strains from China, HKU15-155 and HKU15-44, in the genus *Deltacoronavirus*, when and how this virus was introduced into United States remain unknown. Further investigation is needed to determine whether infection with PorCoV HKU15 results in disease in pigs and if the virus was responsible for the clinical disease observed in outbreaks on the 5 Ohio farms in this study. In addition, surveillance should be conducted in other US states to define the distribution of the virus among the US pig population. Moreover, whole-genome sequence analysis should be performed for other strains from different locations to determine whether the virus was introduced into the United States by a single entry or by multiple entries.
